# Lecithin Exhibits Rapid Contact Toxicity Against *Bemisia tabaci* and Is Associated with Structural and Molecular Responses

**DOI:** 10.3390/insects17090935

**Published:** 2026-09-07

**Authors:** Huinan Xu, Liping Huang, Jiao Du, Jianbin Chen, Xiaobin Shi, Yong Liu

**Affiliations:** 1 Longping Agricultural College, Hunan University, Changsha 410125, China; xhn1663446491@hnu.edu.cn; 2 Yuelushan Laboratory Breeding Program, Changsha 410125, China; hlp1936580786@hnu.edu.cn (L.H.); dujiao1234xy@163.com (J.D.); chenjianbin89@126.com (J.C.); 3 Institute of Plant Protection, Hunan Academy of Agricultural Sciences, No. 726, 2nd Section Yuanda Road, Furong District, Changsha 410125, China

**Keywords:** *Bemisia tabaci*, lecithin, contact toxicity, insect cuticle, transcriptomics, metabolomics

## Abstract

*Bemisia tabaci* is a globally important crop pest and virus vector with widespread resistance to conventional insecticides. We evaluated the insecticidal activity of a soybean-derived lecithin preparation and associated physiological responses. Leaf-surface, dried-residue contact, artificial-diet, and egg bioassays were combined with RNA sequencing, untargeted metabolomics, RT-qPCR, histology, and transmission electron microscopy. Dried-residue exposure yielded a 4 h LC_50_ of 1.16 mg mL^−1^ (95% confidence interval, 0.948–1.403 mg mL^−1^), whereas 10 mg mL^−1^ lecithin caused 100% adult mortality within 4 h and reduced egg hatchability from 99.44% to 37.78%. No mortality was detected after adults were provided with a sucrose diet containing 10 mg mL^−1^ lecithin for 24 h. Exposure at the 4 h LC_50_ was associated with differential expression of 135 genes and changes in 599 metabolic features, including responses related to cuticle organization, lysosomal and autophagy pathways, and amino acid metabolism. Histology and transmission electron microscopy revealed altered abdominal tissue organization and cellular ultrastructure. These findings establish contact-associated and egg-stage activity of lecithin against *B. tabaci* under laboratory conditions and identify accompanying structural, transcriptional, and metabolic responses. The molecular events responsible for mortality and the contribution of ingestion-mediated exposure remain unresolved.

## Simple Summary

Whiteflies damage crops by feeding on plants and spreading viruses, while resistance to many conventional insecticides makes them increasingly difficult to control. We tested whether lecithin, a naturally occurring group of fat-like membrane components widely used in food and biological formulations, could affect adult whiteflies and their eggs. When adult whiteflies contacted tomato leaves or dishes treated with lecithin, mortality increased with concentration and reached 100% at the highest concentration tested. In contrast, adults offered lecithin in a sugar solution did not die during the 24 h test, suggesting that activity depended mainly on surface contact under our conditions. Lecithin treatment also reduced egg hatching from about 99% to 38%. Analyses of gene activity, small molecules, and tissues showed changes involving the insect outer covering and internal cell structures. These findings identify lecithin as a contact-active material against adult whiteflies and eggs under laboratory conditions. Further work is needed to define its active components, determine exactly how it causes injury, and establish its safety and effectiveness on crops and beneficial organisms.

## 1. Introduction

*Bemisia tabaci* is one of the most economically important agricultural pests worldwide due to its extensive host range, rapid reproduction, and ability to transmit numerous plant viruses [1,2]. The Mediterranean cryptic species of *B. tabaci* (MED) has become widely distributed and causes substantial economic losses in vegetable and ornamental crops. Management of *B. tabaci* populations relies largely on chemical insecticides; however, intensive and repeated applications have accelerated the evolution of resistance in major pest populations and compromised long-term field efficacy [1,3,4,5]. These challenges highlight the need for alternative control materials with distinct biological activities and compatible environmental profiles [6].

Natural products represent important sources for discovering new pest management compounds because of their structural diversity and biological activities [7]. However, natural origin alone does not guarantee insecticidal efficacy, target specificity, environmental compatibility, or reduced resistance risk. Therefore, potential candidates require rigorous evaluation through standardized bioassays, chemical characterization, and assessment of effects on both target pests and non-target organisms.

Lecithin is a naturally occurring phospholipid-rich material widely present in plants and biological membranes. Its major components commonly include phosphatidylcholine (PC), together with other phospholipid classes whose relative abundance and fatty-acyl composition vary according to biological source and extraction process [8,9]. The amphiphilic properties of lecithin-derived phospholipids, determined by their hydrophilic head groups and hydrophobic fatty-acyl chains, contribute to their ability to form emulsions, lipid assemblies, and interfacial structures [10,11]. Although lecithin is widely used in food, pharmaceutical, and agricultural formulations, its direct insecticidal activity has received limited attention. Previous studies have shown that individual phospholipid molecules, such as 1-palmitoyl-2-linoleoyl-3-glycerophosphocholine isolated from *Chenopodium ficifolium*, can induce dose-dependent mortality in *Aphis gossypii* [12], suggesting that specific lipid components may possess insecticidal potential. However, whether commercial lecithin formulations exhibit biological activity against *B. tabaci* and how insects respond to such exposure remain largely unexplored.

The insect cuticle is the primary interface between insects and their external environment, providing protection against desiccation, mechanical damage, pathogens, and xenobiotic penetration [13,14]. Because cuticular lipids and proteins contribute to barrier properties, variations in cuticle composition and structure can influence insecticide penetration and susceptibility [15,16]. Exogenous lipid exposure may therefore affect insect surface properties; however, whether lecithin alters cuticle-associated processes in *B. tabaci* remains unknown. Moreover, changes in cuticle-related gene expression after chemical exposure may reflect either direct effects on cuticular integrity or secondary physiological responses associated with stress adaptation and repair.

In addition to structural responses, insecticide exposure can induce complex cellular and metabolic adjustments [17]. Transcriptomic and metabolomic approaches provide complementary information for identifying biological pathways associated with chemical stress, although pathway enrichment alone does not establish direct molecular targets [18]. Integrating multi-dimensional evidence with morphological observations can therefore help generate experimentally testable hypotheses regarding the physiological consequences of insecticide exposure [19,20].

In this study, we systematically evaluated the biological effects of a commercial soybean-derived lecithin preparation against adult and egg stages of *B. tabaci*. Contact toxicity was assessed using leaf-surface and dried-residue assays, while artificial feeding was used to examine detectable effects following dietary exposure. Egg bioassays were performed to determine effects on hatchability. Transcriptomic analysis, untargeted metabolomics, RT-qPCR validation, histological examination, and transmission electron microscopy were then used to characterize molecular, metabolic, and structural responses associated with lecithin exposure. We aimed to define the insecticidal phenotype and identify biological processes associated with exposure without assigning a definitive molecular target or a single mode of action.

## 2. Materials and Methods

### 2.1. Insect Culture and Species Identification

A laboratory colony of *Bemisia tabaci* MED (Mediterranean cryptic species) was maintained on tomato plants (*Solanum lycopersicum*) under controlled environmental conditions (26 ± 1 °C, 70 ± 5% relative humidity, and a 16:8 h light: dark photoperiod). The colony was originally provided by the Institute of Vegetables and Flowers, Chinese Academy of Agricultural Sciences (Beijing, China).

The cryptic species identity was confirmed by mitochondrial cytochrome oxidase I (mtCOI) sequencing. Genomic DNA was extracted from individual whiteflies, and the mtCOI region was amplified using primers C1-J-2195 (5′-TTGATTTTTTGGTCATCCAGAAGT-3′) and R-BQ-2819 (5′-CTGAATATCGRCGAGGCATTCC-3′). The amplified fragment (624 bp) was sequenced and compared with reference sequences available in GenBank. The obtained sequence showed high similarity to the published GenBank reference sequence GQ371165, supporting the identification of the whitefly population as the corresponding cryptic species.

### 2.2. Lecithin Preparation

Soybean-derived lecithin was purchased from MedChemExpress (Monmouth Junction, NJ, USA; Cat. No. HY-B2235; CAS No. 8002-43-5; purity ≥ 98%). Because commercial lecithin is a complex phospholipid mixture rather than a single compound, the detailed proportions of individual phospholipid components, including PC, phosphatidylethanolamine (PE), and phosphatidylinositol (PI), were not available from the manufacturer. The suspension was vortexed for 2 min and sonicated for 30 min at room temperature. Fresh suspensions were prepared for each bioassay to limit time-dependent aggregation.

### 2.3. Biological Bioassays

#### 2.3.1. Leaf-Dip Contact Toxicity Assay

A preliminary leaf-dip assay was performed as described for whiteflies with minor modifications [21,22]. Tomato leaf discs were immersed for 10 s in lecithin suspensions at 0.1, 1, or 10 mg mL^−1^ or in distilled water as the control. After air-drying, tomato leaves were placed in a 9 cm Petri dish and 50 unsexed adults were introduced. Each treatment comprised three independently prepared dishes. Mortality was recorded after 24 h, insects that did not move after gentle probing were scored as dead.

Mortality (%) = (number of dead adults/number of adults exposed) × 100.

#### 2.3.2. Residual-Contact Bioassay and LC_50_ Estimation

To avoid potential interference caused by direct exposure to liquid films, residual-contact toxicity was evaluated using a dried-residue exposure method. For the high-concentration validation, 1 mL of a 10 mg mL^−1^ lecithin suspension was evenly applied onto the entire inner surface of a 9 cm-diameter Petri dish; control dishes received 1 mL of distilled water. The dishes were kept at room temperature for 1 h to allow complete drying (no visible liquid residue). Approximately 50 adult whiteflies were then introduced into each dish, and mortality was recorded after 4 h; individuals showing no movement after gentle stimulation were considered dead.

For the concentration–response assay, the same dried-residue procedure was performed using lecithin suspensions at 0.3125, 0.625, 1.25, 2.5, and 5 mg mL^−1^. Each coated dish was treated as an experimental unit, and each concentration was tested with three independent replicates (dishes). The nominal surface loading (μg cm^−2^) was calculated for each tested concentration as: (C × V × 1000)/A, where C is the suspension concentration (mg mL^−1^), V is the applied volume (1 mL), and A is the total inner surface area of the Petri dish [2 × π × (4.5 cm)^2^].

#### 2.3.3. Artificial-Diet Assay

The potential oral toxicity was evaluated using an artificial feeding system [23,24]. A sterile 15% (*w*/*v*) sucrose solution was prepared in distilled water and filter-sterilized through a 0.22 μm pore-size membrane. Lecithin was incorporated into the sucrose diet at a final concentration of 10 mg mL^−1^ using the vortexing and sonication procedure described above; the control diet consisted of the sucrose solution alone. Approximately 50 adults were introduced into each feeding chamber and allowed to feed for 24 h under standard rearing conditions, after which the number of dead individuals was recorded to calculate mortality. Three independently prepared feeding chambers were used per dietary treatment.

#### 2.3.4. Egg Bioassay

To obtain eggs, tomato plants were exposed to adult *B. tabaci* (approximately 50 adults per plant) for 48 h to allow oviposition. After adults were removed, leaves bearing approximately 60 eggs (counted under a stereomicroscope) were selected. These leaves were completely submerged for 10 s in either a 10 mg mL^−1^ lecithin suspension or distilled water (control), with gentle manual agitation. Following immersion, the leaves were air-dried at room temperature for about 30 min. Each treated leaf was placed individually into a 9 cm Petri dish containing solidified 1.5% water agar (*w*/*v*). The leaf was positioned with its abaxial (lower) surface facing upward and gently pressed onto the agar surface to ensure firm contact, which facilitated continuous hydration of the leaf tissue and prevented egg desiccation. Egg hatchability was assessed after 10 days of incubation, by which time all viable eggs had hatched. An egg was recorded as hatched if its chorion was empty and a first-instar nymph was observed. The initial number of eggs per leaf was recorded prior to treatment, and the number of unhatched eggs was counted at the end of the observation period to calculate the percentage hatch.

Hatchability (%) = (number of hatched eggs/total number of eggs) × 100.

### 2.4. Transcriptomic Analysis

To investigate the molecular responses underlying lecithin-induced toxicity, transcriptomic analysis was performed at the 4 h LC_50_ concentration (1.16 mg mL^−1^) [25,26]. Adult whiteflies were treated with a lecithin suspension at the 4 h LC_50_ concentration, while the control group was simultaneously treated with an equal volume of distilled water; the exposure duration was 4 h. After treatment, only individuals that were alive and exhibited coordinated movement (defined as being capable of walking when gently touched with a fine brush) were collected; moribund and dead individuals were excluded. Approximately 50 adults were pooled per biological replicate, and three independent biological replicates were analyzed for each treatment.

Total RNA was extracted from each sample using TRIzol reagent (Invitrogen, Carlsbad, CA, USA). cDNA library construction and transcriptome sequencing were performed by OE Biotech Co., Ltd. (Shanghai, China). A total of six samples (three biological replicates from the treatment group and three from the control group) were subjected to reference-based transcriptome sequencing, yielding 42.74 Gb of clean data. The clean data volume per sample ranged from 6.76 to 7.36 Gb, with Q30 scores ranging from 92.89% to 93.10% and an average GC content of 41.20%. The clean reads were aligned to the *B. tabaci* reference genome (NCBI Assembly ASM185493v1), and the overall alignment rate across samples ranged from 81.08% to 82.58%.

Based on the alignment results, gene-level read counts were generated using HTSeq (v0.9.1). Differential expression analysis was performed using the DESeq2 R package (v1.40.2) based on raw read counts, with one comparison group (lecithin-treated vs. water-treated controls). Differentially expressed genes (DEGs) were defined by a false discovery rate (FDR)-adjusted *q* < 0.05 and |log_2_ (fold change)| > 1, and a total of 135 DEGs were identified. Gene Ontology (GO) and Kyoto Encyclopedia of Genes and Genomes (KEGG) enrichment analyses of the DEGs were performed using the cluster Profiler R package (v4.4.4), and GO terms and KEGG pathways with an FDR-adjusted *q* < 0.05 were considered significantly enriched (Table S1). The raw transcriptome data have been deposited in the NCBI Gene Expression Omnibus (GEO) under accession number GSE345400.

### 2.5. Metabolomic Analysis

Adult whiteflies (800 per replicate) were exposed to 1.16 mg mL^−1^ lecithin (4 h LC_50_) or distilled water (control) for 4 h, snap-frozen in liquid N_2_, and stored at −80 °C (5 biological replicates per treatment).

For LC-MS, each sample was extracted with 400 µL ice-cold 70% methanol (with internal standard), homogenized (60 Hz, 2 min, −20 °C), sonicated (10 min), incubated (−20 °C, 30 min), and centrifuged (13,000 rpm, 10 min, 4 °C). The supernatant (400 µL) was dried, reconstituted in 100 µL methanol–water (1:4), and filtered. QC samples were pooled from all extracts. Separation used a Dionex U3000 UHPLC with Q-Exactive Plus MS (HESI), on an ACQUITY HSS T3 column (1.8 µm, 2.1 × 100 mm, 45 °C). Mobile phases: 0.1% formic acid in water (A) and acetonitrile (B), gradient 5–100% B in 16 min, flow 0.35 mL min^−1^, injection 10 µL. Full-scan MS (*m*/*z* 100–1000, resolution 70,000) and MS/MS (17,500) with collision energies 10/20/40 eV. Samples randomized; QC injected every 10 runs.

For GC-MS, samples were extracted with 400 µL ice-cold methanol (internal standard), homogenized (60 Hz, 2 min), sonicated (30 min), then added 200 µL chloroform and 400 µL water, sonicated (30 min), incubated (−20 °C, 20 min), and centrifuged (13,000 rpm, 10 min, 4 °C). The upper layer (400 µL) was dried and derivatized with 80 µL methoxyamine-HCl in pyridine (15 mg mL^−1^, 37 °C, 90 min), then 80 µL BSTFA (1% TMCS) and 20 µL n-hexane (70 °C, 60 min). GC-MS was on an Agilent 7890B-5977A with DB-5MS column (30 m × 0.25 mm × 0.25 µm), He 1 mL min^−1^, injector 260 °C, 1 µL splitless. Oven: 60 °C (0.5 min), ramp to 125 °C at 8 °C/min, to 210 °C at 4 °C/min, to 270 °C at 5 °C/min, to 305 °C at 10 °C/min (hold 3 min). Ion source 230 °C, quadrupole 150 °C, 70 eV, full-scan *m*/*z* 50–500, solvent delay 5 min.

LC-MS data were processed with Progenesis QI (5 ppm precursor, 10 ppm fragment, 5% threshold) against HMDB, Lipidmaps, Metlin, and in-house databases. GC-MS data used MS-DIAL and the LUG database. Peak intensities normalized to internal standard. Features with QC-CV > 30% or >50% missing per group were removed; remaining missing values replaced by half of the minimum. Features with score < 36 excluded. PCA and OPLS-DA (R software, 7-fold cross-validation, 200 permutations) were performed; R^2^X, R^2^Y, Q^2^ reported. Differential features were selected by VIP > 1.0, *t*-test *p* < 0.05, and FDR *q* < 0.05. Metabolites without authentic standards were termed “differential metabolic features”. Identified metabolites underwent KEGG enrichment analysis.

### 2.6. RT-qPCR Validation

Eight differentially expressed genes (DEGs) were selected for validation by RT-qPCR. First-strand cDNA was synthesized from total RNA using the HiScript First Strand cDNA Synthesis Kit (Vazyme, Nanjing, China) following the manufacturer’s instructions. RT-qPCR was performed in 20 μL reactions using ChamQ Universal SYBR qPCR Master Mix (Vazyme) on a CFX96 Real-Time PCR Detection System (Bio-Rad Laboratories, Hercules, CA, USA). The thermal cycling program was as follows: 95 °C for 30 s, followed by 40 cycles of 95 °C for 10 s and 60 °C for 30 s, with a subsequent melt-curve analysis to verify amplification specificity. Relative transcript abundance was calculated using the 2^−ΔΔCt^ method, with β-actin as the reference gene. All statistical comparisons for RT-qPCR were performed on ΔCt values as described in Section 2.8; the 2^−ΔΔCt^ values are shown only for visualization. Three independent biological replicates were analyzed per treatment. The primer sequences are listed in Table A1.

In addition to the 4 h validation, the time-dependent expression of *LOC109040128* was examined at 1, 2, 3, 4, and 24 h after exposure to 1.16 mg mL^−1^ lecithin or water control. At each time point, time-matched water-treated controls were included. Expression levels were normalized to the corresponding time-matched control (CK) for each time point. Three independent biological replicates were analyzed per treatment per time point.

### 2.7. Histological and Ultrastructural Observation

Surviving adults after lecithin exposure were collected and fixed for histological examination and transmission electron microscopy.

Samples were fixed in formalin–acetic acid–alcohol (FAA) solution. The fixed specimens were then dehydrated through a graded ethanol series, embedded in paraffin wax, and sectioned at a thickness of 10 μm. The sections were stained with hematoxylin and eosin (H&E) and examined under a ZEISS Axio Observer microscope.

Adult whiteflies were fixed overnight in 2.5% glutaraldehyde in phosphate-buffered saline (0.1 M, pH 7.2) at 4 °C. The specimens were then post-fixed in 1% osmium tetroxide in the same buffer for 2 h at 4 °C. After post-fixation, the samples were dehydrated through a graded ethanol series (30%, 50%, 70%, 80%, 90%, 95%, and 100%), infiltrated, and embedded in epoxy resin. Ultrathin sections (70–90 nm) were cut using an ultramicrotome, stained with uranyl acetate and lead citrate, and examined under a Hitachi HT-7700 transmission electron microscope operating at an accelerating voltage of 80 kV. For each treatment group, ten individuals were processed and examined.

### 2.8. Statistical Analysis

Bioassay data (mortality/hatchability) were analysed by binomial GLM with logit link on raw counts (dead/total or hatched/total), with each dish/leaf as experimental unit. Multiple comparisons used Wald tests with Tukey contrasts; two-group contrasts were extracted from models. All statistical comparisons for RT-qPCR were performed on ΔCt values. For the expression analysis of *LOC109040128*, samples were collected at 1, 2, 3, 4, and 24 h after treatment with lecithin (1.16 mg mL^−1^). At each time point, independent lecithin-treated and water-treated control groups were established. The ΔCt values of the treated group and the corresponding control group at each time point were compared using independent-samples *t*-tests. The 4 h LC_50_ was estimated using probit analysis, with log_10_-transformed concentration (in mg mL^−1^) as the covariate. The model output included the slope coefficient with its standard error (SE), the Z statistic, the Pearson goodness-of-fit χ^2^ statistic, degrees of freedom (df), and the corresponding *p*-value. All tests were two-sided at α = 0.05, using IBM SPSS Statistics 21.0.

## 3. Results

### 3.1. Lecithin Shows Concentration-Dependent Leaf-Surface and Residual-Contact Toxicity

**Figure 1:**
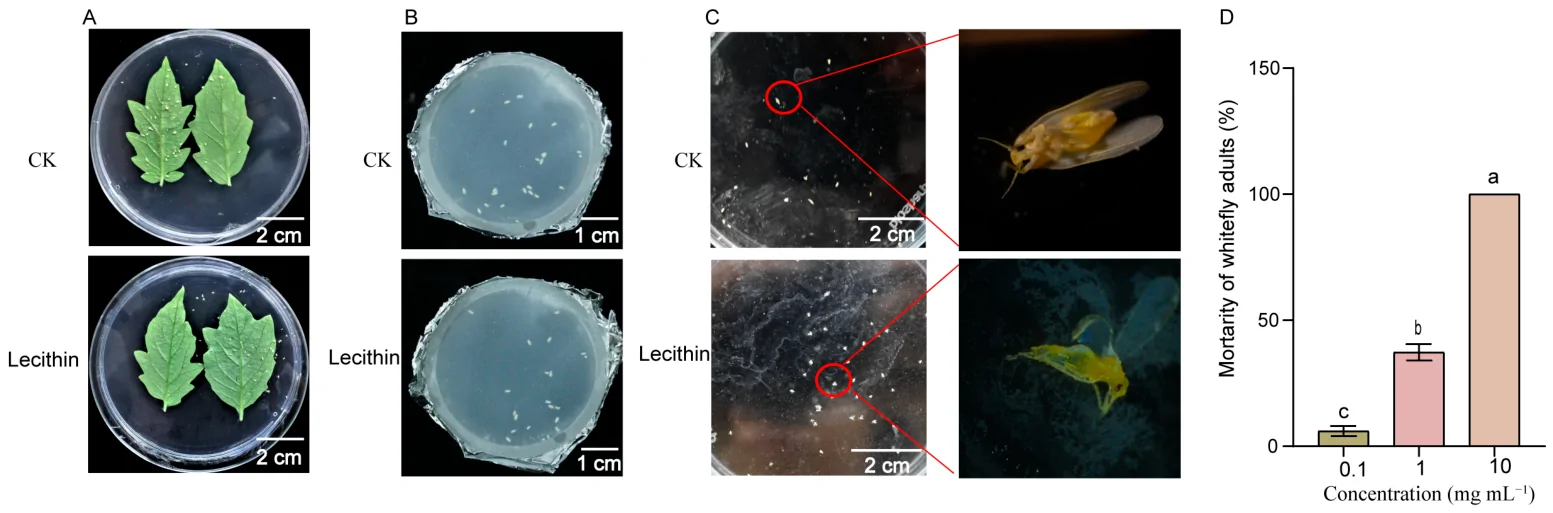
Route and concentration dependent effects of lecithin on adult *Bemisia tabaci*. (**A**) Representative leaf-surface assay images 24 h after adults were exposed to tomato leaf discs treated with water or 10 mg mL^−1^ lecithin. (**B**) Representative artificial-feeding chambers after 24 h access to 15% (*w*/*v*) sucrose diet without or with 10 mg mL^−1^ lecithin; no mortality was detected. (**C**) Representative dried-residue contact assay images 4 h after adults were introduced into dishes treated with water or 10 mg mL^−1^ lecithin. The red circle indicates the region shown in the enlarged image. (**D**) Adult mortality 24 h after leaf-surface exposure to 0.1, 1.0, or 10 mg mL^−1^ lecithin. Bars show mean ± SE (*n* = 3 independently prepared dishes). Mortality was analysed using a binomial GLM with a logit link. Different letters (a, b, c) indicate significant differences among concentrations (*p* < 0.05).

In the preliminary leaf-surface assay, adult mortality after 24 h increased with lecithin concentration, reaching 6.0%, 37.3%, and 100% at 0.1, 1, and 10 mg mL^−1^, respectively (Figure 1A,D). Binomial GLM with logit link on raw mortality counts (dead/total) revealed a highly significant positive effect of concentration on mortality (Wald χ^2^ = 61.12, df = 1, *p* = 0.001; OR = 24.25, 95% CI: 10.90–53.93, per log_10_-unit increase in concentration). The 10 mg mL^−1^ concentration was retained for route-comparison and egg assays, whereas the estimated 4 h LC_50_ was used for molecular and microscopic analyses.

No mortality was detected after adults fed for 24 h on a sucrose diet containing 10 mg mL^−1^ lecithin (Figure 1B). This result indicates no detectable effect under the artificial-diet conditions but does not exclude oral activity because ingestion was not quantified. By contrast, exposure to a dish treated with 10 mg mL^−1^ lecithin caused 100% mortality within 4 h (Figure 1C). Treated adults showed abdominal collapse and conspicuous changes in external appearance.

Probit analysis of the residual contact bioassay data yielded a 4 h LC_50_ of 1.16 mg mL^−1^, with a 95% confidence interval of 0.948–1.403 mg mL^−1^. The fitted regression equation was Probit(p) = −0.136 + 2.159 log_10_(C), where C is the concentration in mg mL^−1^ (log-transformed base 10). The slope coefficient (2.159) had a standard error of 0.243 and was significantly different from zero (Z = 8.892). The model showed good fit (Pearson χ^2^ = 0.345, df = 3, *p* = 0.951) (Table 1 and Table A2). The nominal surface loading corresponding to this LC_50_, calculated based on the total inner surface area of the Petri dish (area = 127.24 cm^2^) and an applied volume of 1 mL, was 9.12 μg cm^−2^.

### 3.2. Lecithin Reduces Egg Hatchability

**Figure 2:**
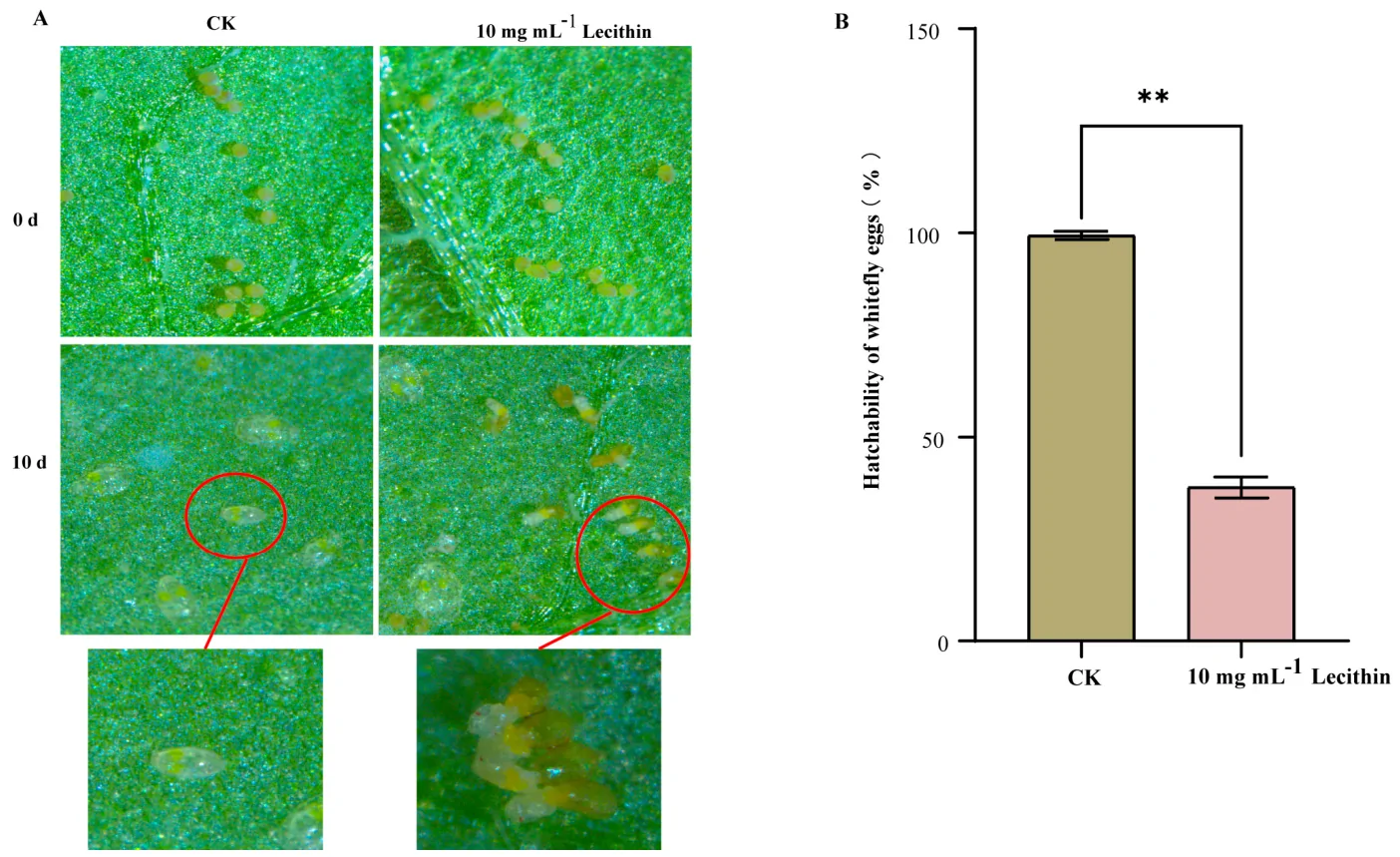
Effect of lecithin on *B. tabaci* egg hatchability. (**A**) Representative eggs immediately after leaf dipping (0 d) and 10 d after treatment with water or 10 mg mL^−1^ lecithin. Enlarged views show a translucent, empty chorion in the water control and retained opaque yellow-to-brown eggs after lecithin treatment. The red circle indicates the region shown in the enlarged image. (**B**) Hatchability at 10 d. Bars show mean ± SE (*n* = 3 independently prepared leaves, each bearing approximately 60 eggs). Statistical inference was performed using a binomial GLM with a logit link. ** indicates significant differences between the lecithin-treated and CK (*p* < 0.01).

Treatment with 10 mg mL^−1^ lecithin significantly reduced egg hatchability from 99.44% in the water control to 37.78%, accompanied by a greater frequency of unhatched, discolored eggs (Figure 2A,B). Binomial GLM with logit link on raw hatchability counts revealed a highly significant treatment effect (OR = 0.0103, 95% CI: 0.0032–0.0335, for lecithin vs. control; *p* < 0.01). These results indicate that lecithin exposure also affects the egg stage; however, the underlying mechanism of hatching failure was not determined in this study.

### 3.3. Transcriptomic Responses to Lecithin Exposure

**Figure 3:**
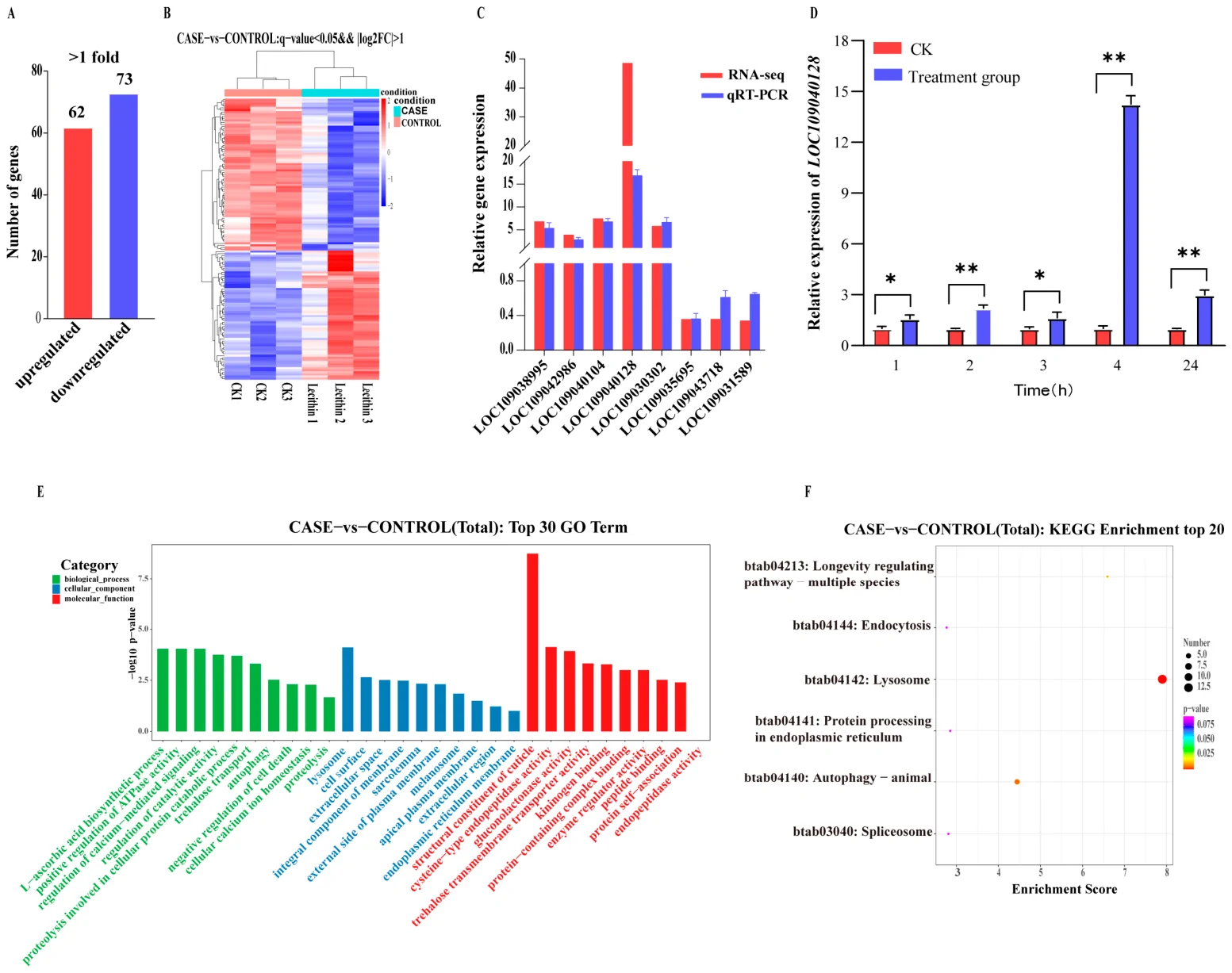
Transcriptomic and RT-qPCR responses of adult *B. tabaci* to lecithin. (**A**) Numbers of differentially expressed genes at *q* < 0.05 and |log_2_ (fold change)| > 1: 62 upregulated and 73 downregulated genes. (**B**) Hierarchical clustering of the 135 differentially expressed genes across three biological replicates per group (CK1–CK3 and Lecithin1–Lecithin3); colors indicate row-scaled expression values. (**C**) RNA-seq fold changes and RT-qPCR validation of eight selected genes. RT-qPCR expression was normalized to *β-actin*; RT-qPCR bars show mean ± SE (*n* = 3 independent biological replicates). (**D**) Time-course expression profile of *LOC109040128* after treatment with 1.16 mg mL^−1^ lecithin or water. Samples were collected at 1, 2, 3, 4, and 24 h post-treatment, with independent water-treated controls included at each time point. Expression levels were normalized to the corresponding time-matched control (CK). Bars represent mean ± SE (*n* = 3 independent biological replicates). * indicates significant differences between the lecithin-treated and corresponding control groups at each time point (independent-samples *t* -test on ΔCt values, *p* < 0.05; ** indicate *p* < 0.01). (**E**) Top 30 enriched Gene Ontology terms. Green, blue, and red bars denote biological process, cellular component, and molecular function, respectively; bar height represents −log_10_(P). (**F**) Displayed KEGG pathway enrichment results. The *x*-axis shows enrichment score, bubble size represents the number of mapped genes, and color represents the enrichment *p* value. CK, water-treated control.

RNA sequencing identified 135 differentially expressed genes in adults exposed to 1.16 mg mL^−1^ lecithin for 4 h, including 62 upregulated and 73 downregulated genes (Figure 3A). Hierarchical clustering separated lecithin-treated and control samples, and the three biological replicates grouped by treatment (Figure 3B). RT-qPCR of eight representative genes showed directionally consistent expression patterns (Figure 3C). GO analysis identified enriched categories that included structural constituent of cuticle and autophagy (Figure 3E). KEGG analysis identified lysosome, autophagy—animal, protein processing in the endoplasmic reticulum, and longevity-regulating pathways among the enriched terms (Figure 3F). These data describe treatment-associated transcriptional responses.

### 3.4. Metabolomic Profiling Shows Broad Treatment-Associated Changes

**Figure 4:**
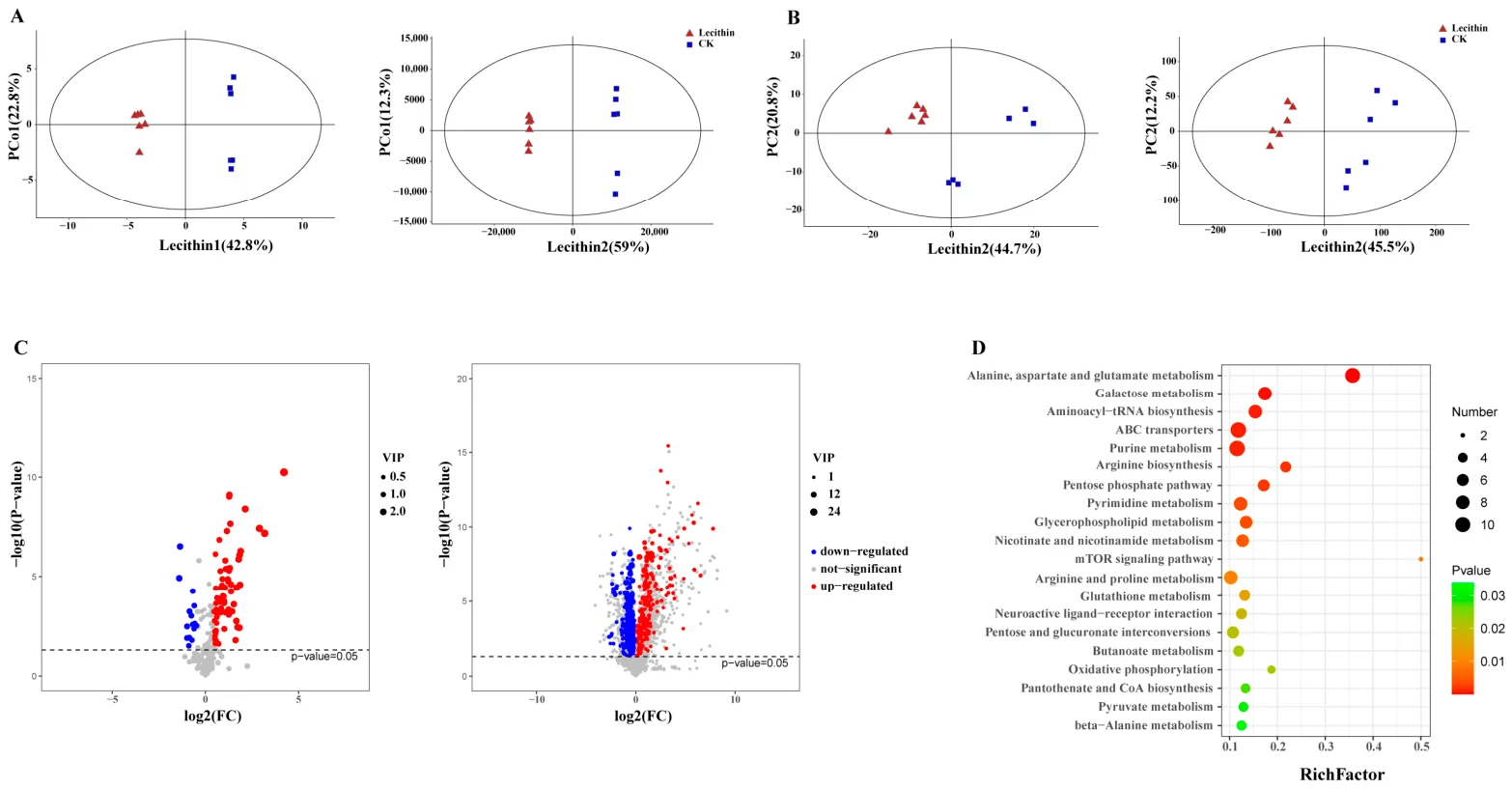
Untargeted metabolomic profiles of adult *B. tabaci* after 4 h exposure to water (CK) or 1.16 mg mL^−1^ lecithin. (**A**) OPLS-DA and (**B**) PCA score plots for GC–MS (left) and LC–MS (right). Red triangles and blue squares represent PC and CK samples, respectively; each symbol is an independent biological replicate (*n* = 5 per treatment per platform). (**C**) Volcano plots for GC–MS (left) and LC–MS (right). Differential metabolic features were screened using the criteria of VIP > 1.0, *p* < 0.05, and FDR-adjusted *q* < 0.05. The size of each point represents its VIP value; red and blue indicate features with increased and decreased abundance, respectively, in the lecithin-treated group relative to the control group, while gray indicates features that did not simultaneously meet all three screening criteria. (**D**) KEGG pathway enrichment of differential features. The *x*-axis shows Rich Factor, bubble size represents the number of mapped features, and color represents the enrichment *p* value. CK, water-treated control.

**Figure A2:**
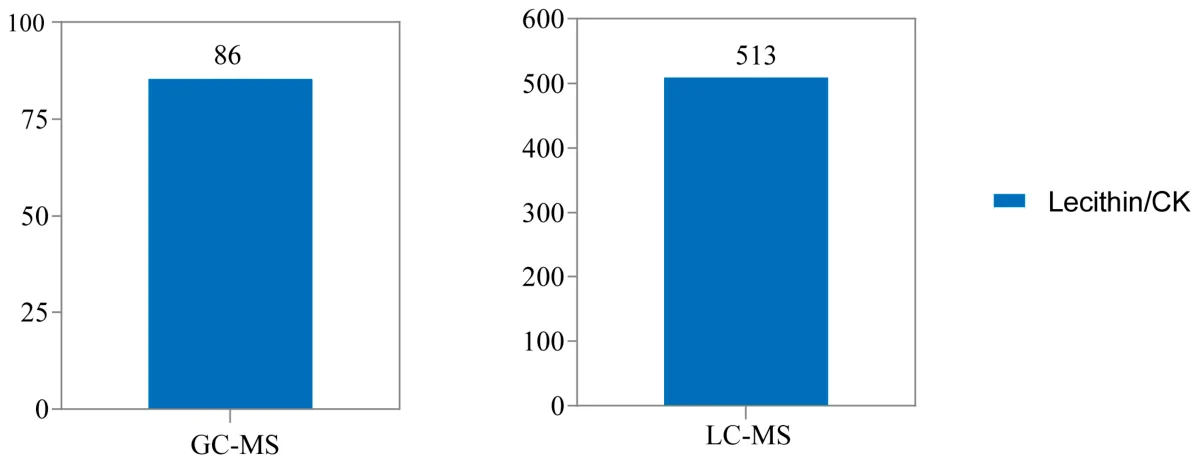
Numbers of differential metabolic features detected after 4 h exposure of adult *B. tabaci* to water (CK) or 1.16 mg mL^−1^ lecithin. The GC–MS (**left**) and LC–MS (**right**) datasets contained 86 and 513 differential features, respectively, using VIP > 1.0 and *p* < 0.05, FDR-adjusted *q* < 0.05 (*n* = 5 independent biological replicates per treatment per platform).

PCA separated the lecithin-treated and control samples in both the LC–MS and GC–MS datasets, with within-group variation also evident (Figure 4B). OPLS-DA likewise separated the two treatments (Figure 4A). Using VIP > 1.0, *p* < 0.05, and FDR-adjusted *q* < 0.05 as the screening criteria, a total of 513 LC-MS and 86 GC-MS differential metabolic features were identified (Figure 4C and Figure A2). In combination, these comprised 250 features with increased abundance and 349 features with decreased abundance after lecithin treatment. The top-ranked KEGG terms included alanine, aspartate and glutamate metabolism, ABC transporters, aminoacyl-tRNA biosynthesis, and several energy- and nucleotide-related pathways (Figure 4D). These patterns indicate broad metabolic change after exposure.

### 3.5. Time-Dependent Expression of LOC109040128

**Figure A4:**
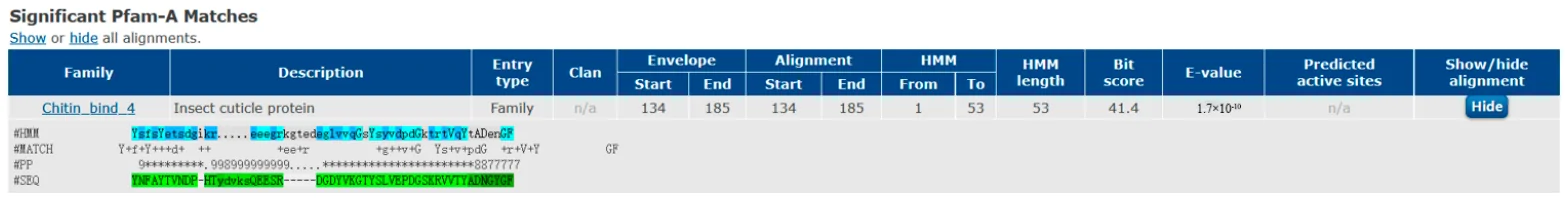
Pfam-A domain match for the predicted LOC109040128 protein. A single significant Chitin_bind_4-family match, annotated as an insect cuticle protein, spans residues 134–185 (HMM positions 1–53 of a 53-residue model), with a bit score of 41.4 and an E value of 1.7 × 10^−10^. The aligned query and profile-HMM sequences are shown below the summary table. In the sequence alignment, * denote fully conserved residues (identical across all sequences), + indicate residues with similar physicochemical properties (partially conserved), and. indicate weakly similar residues.

At all time points examined (1, 2, 3, 4, and 24 h), the expression level of *LOC109040128* in the lecithin-treated group was significantly higher than that in the corresponding time-matched water-treated control group (Figure 3D). The increase was relatively modest during the first 3 h but became pronounced at 4 h, at which the highest mean expression level was observed. Although expression declined at 24 h compared with the peak at 4 h, it remained significantly elevated relative to the corresponding control (*p* < 0.01). Thus, *LOC109040128* exhibited a pronounced temporal response to lecithin exposure, with maximal induction at 4 h. Pfam analysis supported annotation of the encoded protein as an RR-2 cuticular protein (Figure A4).

### 3.6. Histological and Ultrastructural Changes After Lecithin Exposure

**Figure 5:**
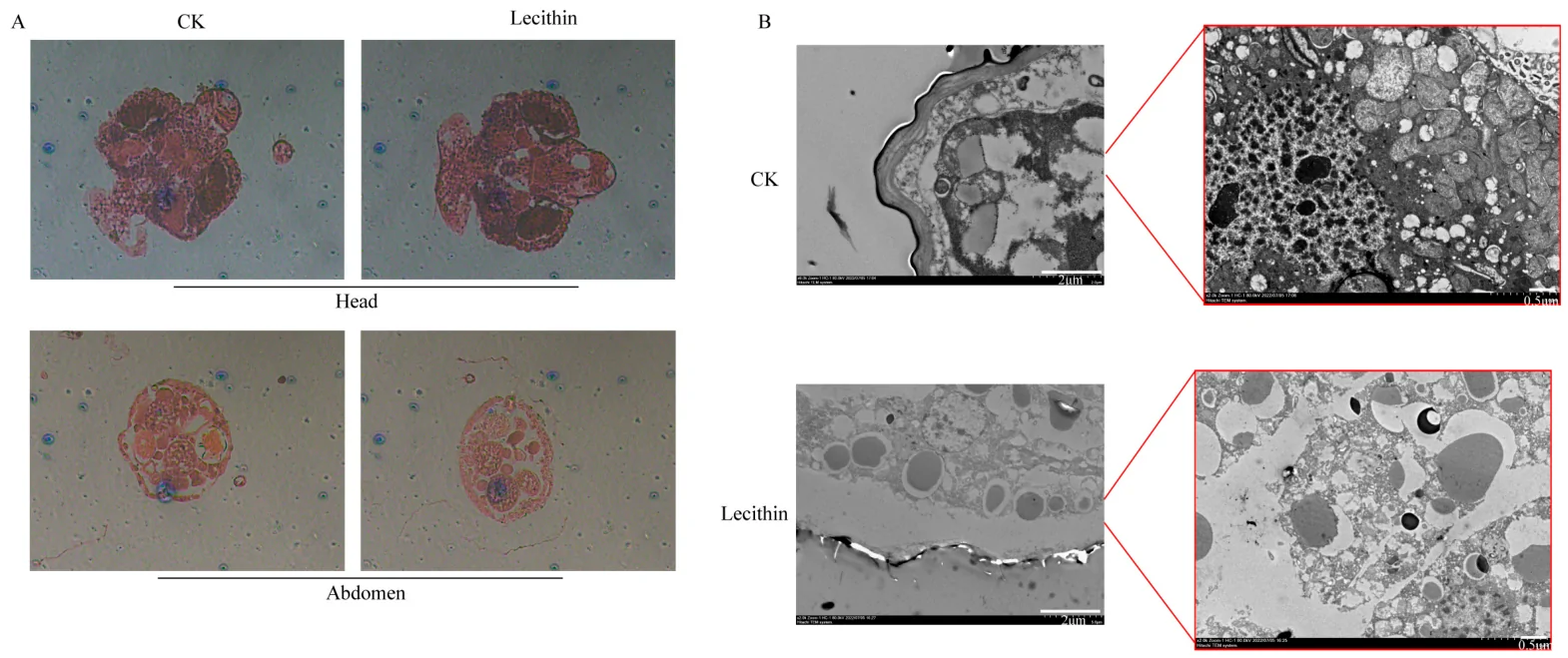
Representative histological and ultrastructural features of adult *B. tabaci* after 4 h exposure to water (CK) or 1.16 mg mL^−1^ lecithin. (**A**) Hematoxylin-and-eosin-stained sections of the head (**upper**) and abdomen (**lower**). (**B**) Transmission electron micrographs of the peripheral cuticular boundary and underlying cells. Low-magnification fields (**left**; scale bars, 2 μm) and corresponding higher-magnification views (**right**; scale bars, 0.5 μm) are shown for CK (**upper**) and lecithin-treated insects (**lower**). CK, water-treated control.

Histological observation of tissue sections revealed that lecithin treatment exerted a limited effect on the overall head morphology of *B. tabaci*, with no significant differences detected in head contour or cellular arrangement between the treated and control groups (Figure 5). In the abdominal region, however, the treated group exhibited increased tissue loosening, with distinct gaps or transparent areas appearing locally, suggesting a decline in tissue integrity, although this did not reach the extent of extensive disintegration or vacuolization. TEM analysis further indicated that, compared with the control group, the lecithin-treated group displayed decreased cytoplasmic electron density and a more loosely structured cytoplasm, accompanied by numerous electron-lucent areas and vacuole-like structures of varying sizes. In some regions, larger cavities were formed, implying a perturbation of the endomembrane system or organelle homeostasis. Additionally, organelle outlines became indistinct with disordered internal architectures, and the outer cellular margins exhibited irregular undulating contours or localized discontinuities. In contrast, the control group maintained continuous boundaries, homogeneous cytoplasm, and regularly distributed organelles.

## 4. Discussion

Our results demonstrate that soybean-derived lecithin exhibits insecticidal activity against *B. tabaci*, with a pronounced dependence on the route of exposure. Lecithin caused rapid and concentration-dependent contact toxicity in adults, with a 4 h LC_50_ of 1.16 mg mL^−1^, and significantly reduced egg hatchability from 99.44% to 37.78%, whereas no detectable oral toxicity was observed in the artificial-diet assay. These findings indicate that the insecticidal activity of lecithin is predominantly associated with contact exposure rather than ingestion. Importantly, the integrated phenotypic, microscopic, transcriptomic, and metabolomic evidence does not support attribution of lecithin toxicity to a single molecular target or a strictly physical mode of action. Instead, the available evidence is more consistent with lecithin-induced alterations at the insect surface accompanied by broader cellular and metabolic stress responses.

The rapid mortality following contact exposure, together with abdominal collapse and abnormalities in external structures, suggests that the insect surface may represent an important interface for lecithin activity. The persistence of toxicity after the treated surfaces had dried further indicates that mortality cannot be explained solely by prolonged immersion in a liquid film. Similar rapid contact toxicity has been reported for petroleum-derived oils, which can interact with the insect surface and subsequently affect internal tissues and physiological processes [27]. Given the amphiphilic nature of lecithin, interactions between lecithin and lipid-rich components of the insect surface may contribute to its contact activity [28]. However, the present data cannot determine whether such interactions directly impair the surface barrier or instead initiate secondary physiological responses. Therefore, the surface alterations observed in this study should be regarded as evidence of a response associated with lecithin exposure rather than direct evidence of primary cuticular disruption.

The absence of detectable oral toxicity further highlights the importance of exposure route in lecithin toxicity. A similar difference between contact and ingestion toxicity has been reported for mineral oils, for which topical exposure resulted in substantially greater mortality than dietary exposure [29]. This difference may reflect the physicochemical properties of these compounds, their localization at the insect–environment interface, or limited bioavailability following ingestion. It is also possible that lecithin, when mixed with sucrose in the artificial diet, undergoes changes in physical dispersion or forms micellar structures that reduce its availability at potential target sites. However, we acknowledge that we did not directly characterize the physicochemical state of lecithin in the feeding solution, nor did we quantify the amount actually ingested by individual whiteflies. These possibilities therefore remain speculative and require further investigation. Although concentration-dependent physiological effects, including hormetic responses, have been reported for some petroleum-derived oils, whether lecithin produces comparable responses remains unknown [30,31,32,33]. Thus, distinguishing contact-specific activity from intrinsic systemic toxicity will be important when evaluating the insecticidal potential of lecithin.

Lecithin also significantly reduced egg hatchability, indicating that its biological activity is not restricted to the adult stage. The reduction in hatchability may result from direct effects on the egg surface, altered chorion permeability, or impaired embryonic development [34,35,36]. However, the present data cannot distinguish among these possibilities. Future studies measuring chorion permeability, water loss, embryonic viability, and developmental progression will be necessary to determine whether the egg surface represents an important site of lecithin action. The differential responses observed between developmental stages also suggest that lecithin may affect distinct biological processes in eggs and adults rather than acting through a single conserved mechanism throughout development.

Histological and transmission electron microscopy observations provided further evidence of tissue-level responses following lecithin exposure. Treated adults exhibited less compact abdominal tissues, reduced cytoplasmic electron density, increased vacuolation, and disorganization of intracellular structures. These alterations are consistent with cellular stress and impaired tissue homeostasis. Similar tissue-level effects have been reported following exposure to petroleum-derived oils, which can accumulate in lipid-rich tissues and interfere with physiological processes [27]. However, microscopic abnormalities alone cannot determine whether membrane damage represents a primary event or a consequence of downstream stress responses. Thus, the present microscopic observations support the occurrence of cellular and tissue injury following lecithin exposure but do not establish a direct membrane-disruptive mechanism.

The transcriptomic and metabolomic analyses further indicated that lecithin exposure induced a broad physiological response. Differentially expressed genes were significantly enriched in pathways related to lysosomal function, autophagy, endoplasmic reticulum protein processing, and longevity-associated processes, whereas metabolomic changes involved amino acid metabolism, ABC transporter-related processes, and energy metabolism. Collectively, these responses suggest activation of cellular stress, protein processing, transport, and metabolic regulatory processes following lecithin exposure. Of particular interest, the RR-2 cuticular protein gene *LOC109040128* was strongly and transiently induced, accompanied by significant enrichment of cuticle-associated functional categories. This response is consistent with involvement of the cuticular system in the physiological response to lecithin exposure [28]. However, the transcriptomic and metabolomic data were obtained at a single concentration (LC_50_) and a single time point (4 h), which limits our ability to distinguish adaptive responses from pathological changes or to infer causal relationships. The induction of cuticle-associated genes, for instance, could represent a protective compensatory response, a secondary consequence of tissue injury, or a non-specific stress reaction. Functional validation through RNA interference, combined with measurements of cuticular permeability and water loss, will be required to determine whether changes in cuticle-associated gene expression functionally contribute to lecithin susceptibility.

Several limitations should be considered when interpreting these findings. First, the commercial soybean-derived lecithin used in this study is a complex mixture, and the relative contributions of individual phospholipid components to its insecticidal activity remain unclear. Second, transcriptomic and metabolomic analyses were performed at a single concentration and time point, limiting our understanding of the temporal dynamics of the physiological response. Third, the microscopic observations were primarily qualitative and should be complemented by quantitative measurements of tissue integrity and cellular damage. Finally, the insecticidal efficacy observed under laboratory conditions cannot be directly extrapolated to field performance. Previous studies of mineral oils have shown that their distribution and persistence on or within plants can influence their biological activity [37]. Therefore, the stability, surface behavior, and persistence of lecithin under realistic application conditions should be evaluated. In addition, because mineral oils can interfere with virus retention and transmission in aphids [38], future studies could investigate whether lecithin has similar effects on virus–vector interactions.

In conclusion, soybean-derived lecithin exhibits rapid and predominantly contact-mediated insecticidal activity against adult *B. tabaci* and significantly reduces egg hatchability, whereas no detectable oral toxicity was observed under the conditions tested. The combined morphological, transcriptomic, and metabolomic evidence indicates that lecithin exposure is associated with alterations in the insect surface and subsequent cellular and metabolic stress responses. However, the available evidence is insufficient to identify a single molecular target or to support a strictly physical mode of action. Whether lecithin can be developed into a practical pest management tool will depend on future research in formulation optimization, field application technology, and non-target safety assessment.

## 5. Conclusions

Lecithin exhibited concentration-dependent contact toxicity against adult *B. tabaci* with a 4 h LC_50_ of 1.16 mg mL^−1^, and significantly reduced egg hatchability under laboratory conditions, whereas no oral toxicity was detected. Exposure was associated with cuticular disorganization, ultrastructural cellular damage, and broad transcriptional and metabolic stress responses, including enrichment of autophagy, lysosomal, and cuticle-related pathways. These results identify lecithin as a contact-active phospholipid preparation in this assay system; however, the current data do not establish a direct molecular target or a definitive mode of action. The observed omics changes are hypothesis-generating and require functional validation to distinguish adaptive responses from causal mechanisms. Future research should address formulation stability, quantitative exposure assessment under field conditions, and non-target organism safety to comprehensively evaluate the practical potential of lecithin as a sustainable pest management tool.

## Figures and Tables

**Table 1:** Probit regression parameters and 4-h LC_50_ of lecithin against adult *Bemisia tabaci* in a residual contact bioassay.

Regression Equation (mg mL^−1^)	4 h LC_50_ (mg mL^−1^)	95% CI	*p*	χ^2^	df	Z	Slope SE
Probit(p) = −0.136 + 2.159 log_10_(C)	1.16	0.948–1.403	0.951	0.345	3	8.892	0.243

LC_50_ denotes the concentration estimated to cause 50% mortality after 4 h; CI, confidence interval.

**Figure A1:**
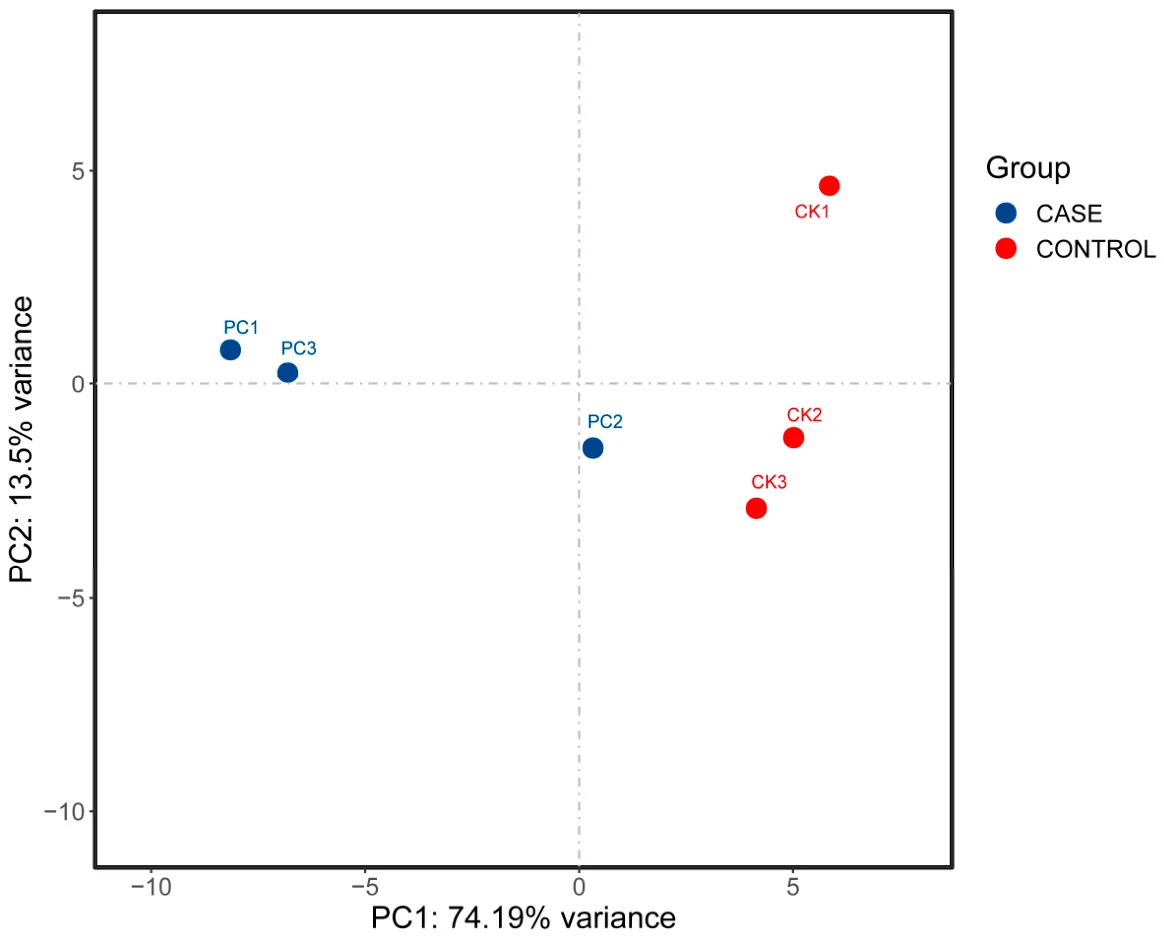
Principal component analysis (PCA) score plot of metabolomic profiles of adult *Bemisia tabaci* after 4 h exposure to water (control) or lecithin at the 4 h LC_50_ concentration (1.16 mg mL^−1^). PC1 and PC2 explained 74.19% and 13.5% of the total variance, respectively. Each symbol represents an individual biological replicate (*n* = 3 per group). Blue circles represent control samples; red squares represent lecithin-treated samples. The two groups were clearly separated along PC1, indicating a distinct metabolic response to lecithin exposure.

**Figure A3:**
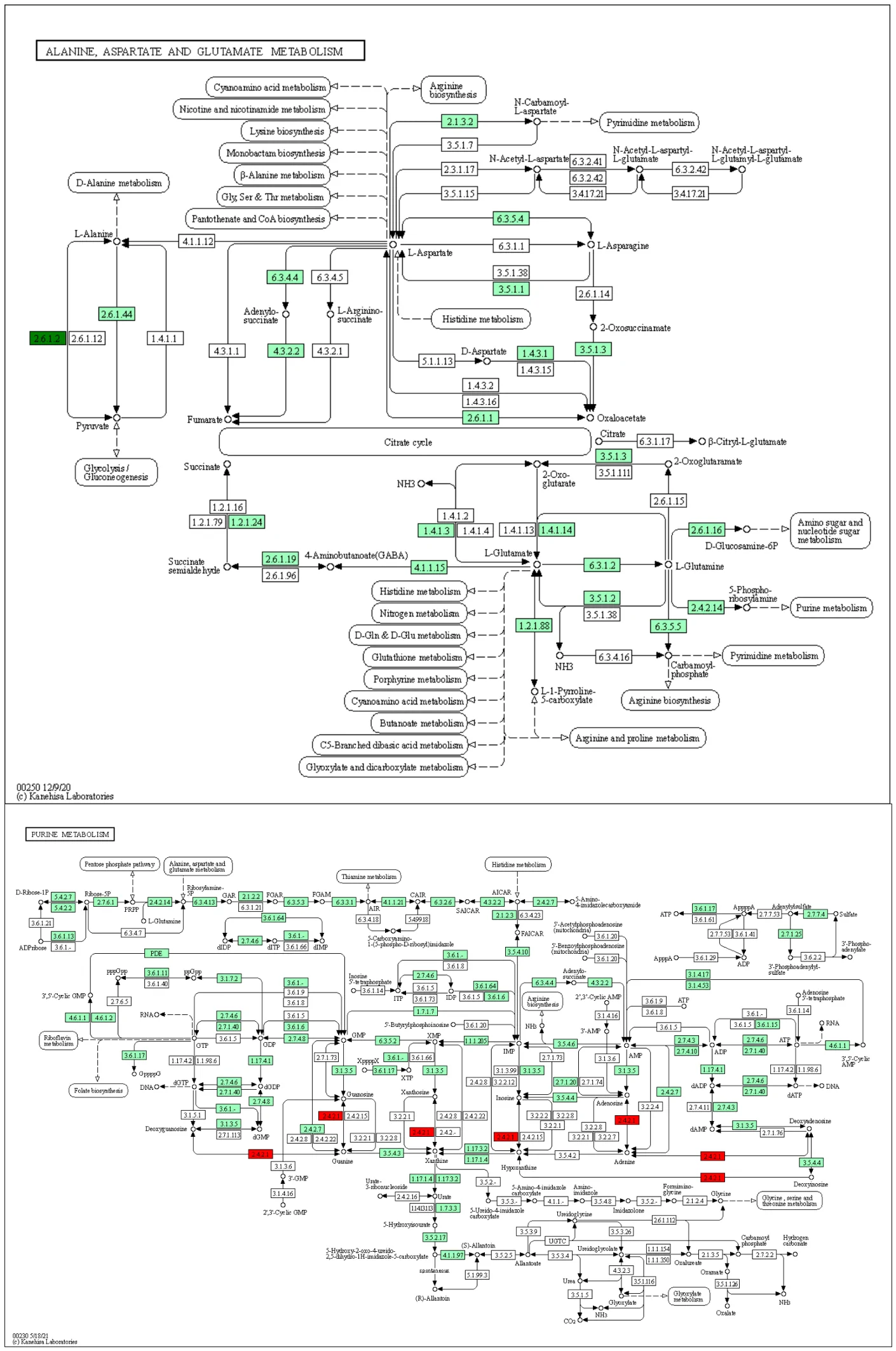
Integrated KEGG pathway overlays for alanine, aspartate and glutamate metabolism (**up**) and purine metabolism (**down**) after 4 h exposure of adult *B. tabaci* to water or 1.16 mg mL^−1^ lecithin. Transcriptomic changes are represented by the blue–white–red scales and metabolomic changes by the red–white–green scales displayed above each map; the scale limits are pathway-specific. The overlays combine RNA-seq data (*n* = 3 independent biological replicates per treatment) with untargeted metabolomic data (*n* = 3 independent biological replicates per treatment per platform).

## Data Availability

The original contributions presented in this study are included in the article/Supplementary Material. Further inquiries can be directed to the corresponding authors.

## Supplementary Materials

The following supporting information can be downloaded at https://www.mdpi.com/article/10.3390/insects17090935/s1, Table S1.

## Abbreviations

The following abbreviations are used in this manuscript:

ANOVA	analysis of variance
CI	confidence interval
CK	water-treated or untreated control, as specified
GC–MS	gas chromatography–mass spectrometry
GO	Gene Ontology
KEGG	Kyoto Encyclopedia of Genes and Genomes
LC–MS	liquid chromatography–mass spectrometry
LC_50_	median lethal concentration
OPLS-DA	orthogonal partial least-squares discriminant analysis
PC	lecithin-treated group label used in the metabolomic figures
PCA	principal component analysis
RNA-seq	RNA sequencing
RT-qPCR	reverse-transcription quantitative PCR
SE	standard error
VIP	variable importance in projection

## Appendix A

**Table A1:** Primers used for RT-qPCR.

Gene Name	Purpose	Melting Temperature	Primer Sequence (5′-3′)
*β-Actin*-F	*B. tabaci* reference genes	60 °C	CGCTGCCTCCACCTCATT
*β-Actin*-R			ACCGCAAGATTCCATACCC
*LOC109038995*-*qF*	*LOC109038995* qPCR	60 °C	CGTACCCAGTTGACGTACCC
*LOC109038995*-*qR*			GGTACGGCTTAGGGATGGTG
*LOC109042986-qF*	*LOC109042986* qPCR	60 °C	GATCTTCCGGAACACACGGT
*LOC109042986*-*qR*			GCCGTTGACCGTTCCATTTC
*LOC109040104*-*qF*	*LOC109040104* qPCR	60 °C	CGACCCTTACTACGACCCC
*LOC109040104* *-qR*			GTCTGGATTCGTGCTGGGAC
*LOC109040128*-*qF*	*LOC109040128* qPCR	60 °C	ACAACTTCGCCTACACCGTC
*LOC109040128*-*qR*			CCGTCAGGTTCGACTAACGA
*LOC109035695* *-qF*	*LOC109035695* qPCR	60 °C	TTTATGCAAACGCCATCACCAG
*LOC109035695* *-qR*			AAGTGGCTTCAAAGAGAGGCT
*LOC109043718* *-qF*	*LOC109043718* qPCR	60 °C	GCTCCATGCATCCCAGAACA
*LOC109043718* *-qR*			AGTGGTGTCATACCCCTCGAA
*LOC109031589*-*qF*	*LOC109031589* qPCR	60 °C	TCTTCGGAAAGAGCACCCAG
*LOC109031589*-*qR*			CGCGAGAATGGAGGAGACAA
*LOC109038302-qF*	*LOC109038302* qPCR	60 °C	ACTTCGCCTACAAGAGCGTC
*LOC109038302-qR*			GATGATGGGCTTGGCAGAGT

**Table A2:** Four-hour residual-contact toxicity of lecithin to adult *Bemisia tabaci*.

Concentration (mg mL^−1^)	Mortality (Mean ± SE) ^i^	Total Individuals
0.3125	5.67 ± 0.33	50
0.625	15 ± 0.58	50
1.25	25 ± 2	50
2.5	38 ± 1	50
5	46.33 ± 0.33	50

^i^ Values are means ± standard error (SE) of three independent biological replicates.
